# Sequencing of complete mitochondrial genome of brown algal *Saccharina* sp. ye-C

**DOI:** 10.1080/23802359.2017.1298414

**Published:** 2017-04-11

**Authors:** Linhong Teng, Dong Xu, Xiaowen Zhang, Benxin Wang, Naihao Ye

**Affiliations:** a Yellow Sea Fisheries Research Institute, Chinese Academy of Fishery Sciences, Qingdao, China;; b Shandong Hongye Foods Co., Ltd, Rongcheng, China;; c Function Laboratory for Marine Fisheries Science and Food Production Processes, Qingdao National Laboratory for Marine Science and Technology, China

**Keywords:** *Saccharina* sp. ye-C, complete mitochondrial genome, Illumina sequencing

## Abstract

The complete sequence (37,609 bp) of the mitochondrial DNA (mtDNA) of the *Saccharina* sp. ye-C was determined using Illumina sequencing data. The genome contains 38 protein-coding genes (PCG), 3 ribosomal RNA (rRNA), 25 transfer RNA (tRNA) genes that are typical of *Saccharina* mtDNA. The phylogenetic analysis based on the mitochondrial genomes of brown algae showed that *Saccharina* sp. ye-C formed a robust clade with *Saccharina coriacea*, which strongly supports their close phylogenetic relationship.

The kelp *Saccharina* are large seaweeds with two distinctive generations of microscopic gametophytes and large elaborate sporophytes (Kawai et al. 2016). They belong to the brown algae (Phaeophyceae) in the order *Laminariales*. *Saccharina japonica* is an important economic alga which has been widely cultivated in China, Japan and Korea (Wang et al. 2013). However, in China *S. japonica* suffered close breeding for generations which have caused significant issues such as loss of genetic varieties, decreasing growth rate and massive mortality (Guan et al. 2016). Recently, the genome of *S. japonica* has been sequenced (Ye et al. 2015), which could strongly promote the genetic improvement of the species. In the study, genomes of several strains of *Saccharina* collected from different locations all over the world were re-sequenced. Under this background, we have enough Illumina sequencing data to recover some mitochondrial genomes of the re-sequenced strains to develop more reliable genetic tools. In this study, the complete mitochondrial genome of a wild strain (NO. KT315643), sampled in Sakhalin in Russia is recovered and named *Saccharina* sp. ye-C based on the phylogenetic analysis with 16 complete brown algae mitochondrial genomes.

Modified phenol-chloroform procedure was used to extract the total genomic DNA (Greco et al. 2014). 4G PE100 reads in total was produced by the second-generation Illumina sequencing.

42,452 reads were mapped to the *S. japonica* mitochondrial genome (37,609 in length) using Genious (http://www.genious.com) and no gap region was found in the mapping result. BLAST (Altschul et al. 1997), DNAstar (Burland 2000), and DOGMA (http://dogma.ccbb.utexas.edu) were employed to annotate the mitochondrial genome and the annotated file was submitted to NCBI using Sequin (http://www.ncbi.nlm.nih.gov/projects/Sequin/).

The length of complete *Saccharina* sp. ye-C is 37,609 bp and the genome contains 38 protein-coding genes (*rps2-4*, *rps7-8*, *rps10-14*, *atp6*, *atp8*, *atp9*, *cox1-3*, *nad1-7*, *nad9*, *nad11*, *nad4L*, *rpl2*, *rpl5*, *rpl6*, *rpl14*, *rpl16*, *rpl19*, *rpl31*, *ORF41*, *ORF130*, *ORF377*, *tatC* and *cob*), 25 transfer RNA (tRNA) genes, 3 ribosomal RNA (rRNA) genes (*5S* rRNA, *16S* rRNA and *23S* rRNA). All 38 protein-coding genes (PCGs) have typical initiation codons (ATG). The numbers of PCGs that have complete termination codons TAA, TAG, TGA are 27, 7 and 4, respectively. No incomplete stop codons were found. The overall GC content is 35.35%, which is well within the normal range of brown mitochondrial DNAs. Nucleotide frequency of the H-strand is as follows: T, 36.29%; A, 28.36%; C, 14.74%; and G, 20.61%. The mitogenome of *Saccharina* sp. ye-C encodes 9644 amino acids, excluding the stop codons. All the 25 typical tRNAs, ranging from 71 to 88, possess a complete clover leaf secondary structure. The rRNAs of the *5S* rRNA, *16S* rRNA and *23S* rRNA genes are 133 bp, 1535 bp and 2736 bp in length, respectively.

Phylogenetic analysis based on other 16 brown algae complete mitochondrial sequence data show that *Saccharina* sp. ye-C belongs to a *Saccharina* clade and is closely related to *S. coriacea*. The result was consistent with recent phylogenetic analyses and certain morphological characters (Zhang et al. 2013; Xu et al. 2016). Complete mitochondrial genomes have enhanced the resolution and statistical confidence of inferred phylogenetic trees (Figure 1).

**Figure 1.**
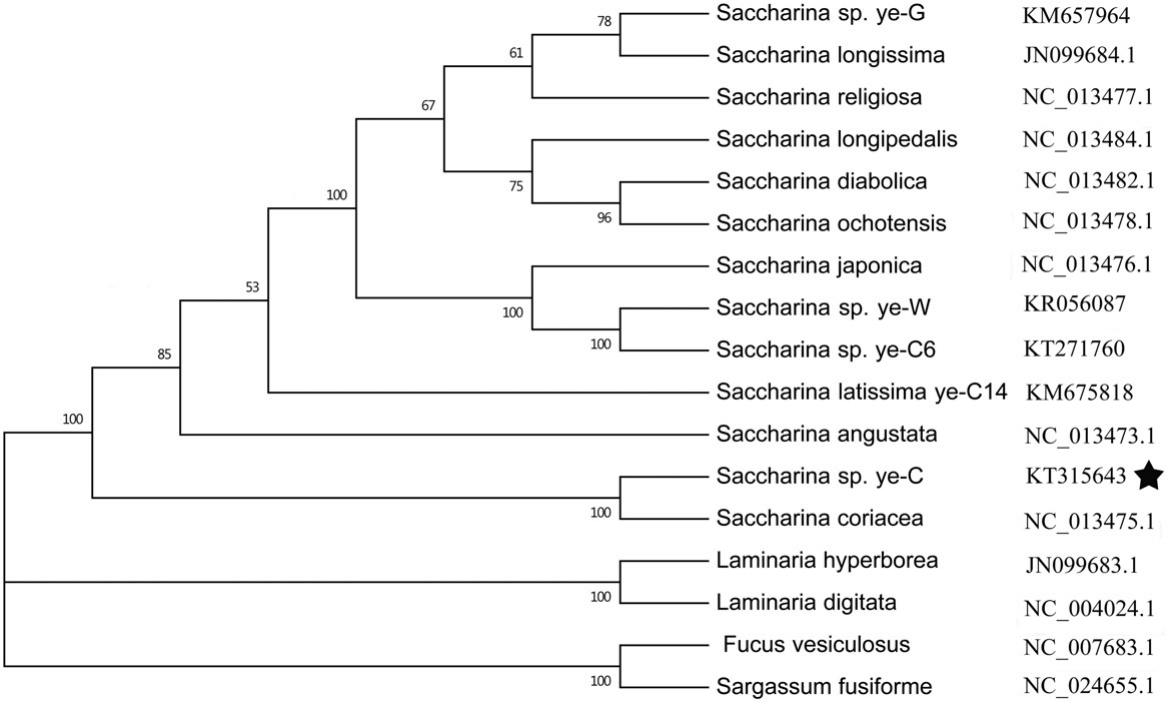
Phylogenetic tree of ML analyses based on complete mitochondrial nucleotide acid sequences of 17 brown algae. Pentagrams stand for the species studied in this work.
